# Summer Shoes

**Published:** 1873-05

**Authors:** 


### SUMMER SHOES.

The old-fashioned "pumps" are best for wear from May until November. The ankles are strengthened by being made to rely on themselves, as it were; for if they are supported by the ordinary boot, they soon look for that support, and, when unsupported, are easily sprained or dislocated.

The ordinary boot keeps the whole foot in a kind of steam-bath all the time, and must necessarily debilitate the skin and impair the vigor of the circulation, thus laying the foundation for cold feet and the multitude of ailments engendered thereby.

But whether pumps or boots are worn during warm weather, the feet should be washed in tepid water every night, and in cold water every morning, wiped thoroughly dry in both cases.

